# The Modification of Vital Signs According to Nursing Students’ Experiences Undergoing Cardiopulmonary Resuscitation Training via High-Fidelity Simulation: Quasi-Experimental Study

**DOI:** 10.2196/11061

**Published:** 2018-08-15

**Authors:** David Fernández-Ayuso, Rosa Fernández-Ayuso, Cristino Del-Campo-Cazallas, José Luis Pérez-Olmo, Borja Matías-Pompa, Josué Fernández-Carnero, Cesar Calvo-Lobo

**Affiliations:** ^1^ San Juan De Dios' University School of Nursing and Physical Therapy Universidad Pontificia Comillas Madrid Spain; ^2^ Emergency Medical Services Nursing Department SUMMA 112 Madrid Spain; ^3^ Health Science Institute Department of Health Sciences Rey Juan Carlos University Madrid Spain; ^4^ Research Multidisciplinary Group for Treatment of Pain URJC-Banco Santander Excellence Research Group Madrid Spain; ^5^ Physical Therapy, Occupational Therapy, Rehabilitation and Physical Medicine Department Rey Juan Carlos University Madrid Spain; ^6^ Institute of Biomedicine Department of Nursing and Physical Therapy Universidad de León Ponferrada Spain

**Keywords:** high-fidelity simulation training, nursing students, vital signs, stress, anxiety.

## Abstract

**Background:**

High-fidelity simulation represents a primary tool in nursing education, especially when hands-on practical training is involved.

**Objective:**

We sought to determine the influence of high-fidelity clinical simulation, applied during cardiopulmonary resuscitation (CPR) training, on blood pressure, heart rate, stress, and anxiety levels in 2 groups of nursing students. One group had experience in health contexts, whereas the other group had none.

**Methods:**

We performed a quasi-experimental study. Data were collected between May and June 2015 and included measurements of all the resting values, before and after participation in CPR clinical simulations regarding the 2 groups of university students (ie, with and without experience).

**Results:**

An increase in vital signs was observed in students after participating in a clinical simulation scenario, especially the heart rate. In all students, increased stress and anxiety levels were observed before the first simulation case scenario. Also, in all study groups, a decrease in vital signs, stress levels, and anxiety was observed throughout the study.

**Conclusions:**

Participation in high-fidelity simulation experiences has both physiological and psychological effects on students.

## Introduction

Over recent years, the value of structured training for education in cardiopulmonary resuscitation (CPR) has increased. Furthermore, according to the European Resuscitation Council (ERC), the probabilities of survival depend on 3 related factors: the application of the chain of survival, updates on recommendations and guidelines, and training in vital support techniques [1]. In this sense, the ERC set down the basis for CPR training in their recommendations made in 2010 [2], placing a special emphasis on the fact that training programmes should adapt to the demands of the environment, to the role of those who intervene, and to the probability of a specific event taking place. Nonetheless, and as acknowledged by the ERC, certain gaps remain in the evidence surrounding educational models for the acquisition and maintenance of competencies in advanced cardiopulmonary resuscitation [3]. In parallel, the training of health professionals in CPR has undergone an exponential increase over the last twenty years, due to new teaching methods based on high-fidelity clinical simulation (HFCS). This is defined by the International Nursing Association for Clinical Simulation and Learning as the context in which simulation takes place and which can vary in length and complexity, according to the objectives sought [4]. The teaching methods involved vary widely, as they can be based on the use of basic simulators for airway training, vascular canalization, and electrical therapies, or involve the development of high-reality or high-fidelity simulation environments. Thus, students can benefit from key features, such as the possibility of integrating all the nursing competencies related to caring for critical patients (ie, teamwork, leadership, communication, reflection,) within a safe and realistic environment. This may be beneficial both for students as well as for patients by avoiding confidentiality problems, shortening learning times, and increasing the level of self-confidence and satisfaction. This also enables the re-creation of rare, but critical, pathological processes, the application of protocols, and the structured evaluation of competencies in critical patients [5-9].

Clinical simulation scenarios, as a teaching method employed in the training of health professionals, facilitate objective teaching models, which otherwise would be limited by ethical, social, administrative, and legal parameters [10]. Nonetheless, simulation-based education presents several disadvantages, including the difficulty faced by certain students when they are to face real clinical contexts appropriately as they often treat patients distantly, or as if it were a game. Furthermore, this approach requires more significant investments, equalling a greater burden for students and teachers, and may have unwanted emotional effects on students, producing anxiety and stress, which have negative correlations for the results of CPR training. Also, several studies have described a lack of confidence, transfer of experienced emotions to the simulation environment, as well as a lack of evidence compared with traditional methods [7,11-14].

Previously, critical care residents under HFCS training increased their heart rate (HR), although not their blood pressure (BP), and their state anxiety was maintained under low anxiety levels, showing differences between men and women [15]. To the authors’ knowledge, there is a lack of research addressing the vital sign changes according to student nurses’ experience undergoing cardiopulmonary resuscitation training via HFCS. Our objective, based on our research on stress during high-fidelity simulation [14], was to determine the influence of this methodology on physiological parameters (ie, BP and HR) as activators of the sympathetic autonomous nervous system, as well as on psychological aspects (ie, stress and anxiety), in 2 groups of nursing students (with and without experience) in a CPR training programme. Our working hypothesis was that the participation of nursing students in high-fidelity simulation scenarios increases the levels of stress, anxiety, and vital signs for all types of health care students, as well as for those with, and without, experience.

## Methods

### Design

A quasi-experimental study was performed with measurements of all the variables at baseline, before, and after participation in standardized clinical simulation experiences during the 2014 academic year at the Comillas Pontifical University (San Juan de Dios School of Nursing and Physical Therapy of Madrid, Spain)*.* The students were divided into 2 groups: one group had experience in health contexts, while the other group did not.

### Participants

The sample comprised 107 students out of a total of 120 who were enrolled in the subject “Physiopathology of the Critical Patient and Vital Support.” Participation in the study was voluntary, and all students were informed of the educational nature of the research, including the fact that this would not affect the course evaluation. The inclusion criteria consisted of students enrolled in the previously cited subject and who were in their second year of the nursing degree program taught by the Comillas Pontifical University according to the current recommendations of the European Council in Resuscitation during the 2014 academic year [2]. We excluded students who had taken more caffeine than the average (in relation to the effect on their resting values) or who were under the effects any drugs affecting the central nervous system or the cardiovascular system (benzodiazepines, antidepressants, beta-blockers or flu vaccines), as well as those who did not wish to participate [14].

### Ethical Considerations

All students signed the informed consent for participating in the research study. Furthermore, the anonymity of participants was guaranteed, as well as that of the data obtained and its safeguarding. Before commencement of the study, the corresponding ethical-legal permissions for performing the study were obtained, as well as the official approval of the project on behalf of the Research Committee from the Nursing School of the Comillas Pontifical University.

### Data Collection

The research took place at the Unit of High-fidelity Simulation in Nursing Care at the University School of Nursing and Physiotherapy. For both the invasive procedures and for providing immediate feedback to students regarding their interventions, a mannequin capable of simulating physiological responses was used. The study was performed during the 2014 academic year, with data collection taking place between May and June 2015. It was planned in 3 successive phases as described in the following.

**Phase II physiological and psychological baseline parameters.**

- Heart Rate (HR) and Blood Pressure (BP): For all students, the basal parameters of HR and BP were measured via 3 measurements, calculating the mean with a certified and approved tensiometer (Omrom M3-HEM-7200-E2; Omron Health care Europe BV, Hoofddorp, Netherlands) and a Nonin Onyx Vantage 9590 pulsioximeter (Nonin Medical, Inc, Plymouth, MN, USA), following the recommendations of the European Hypertension Society [18].
- Anxiety: This was determined by using the state-trait anxiety questionnaire by Spielberger [19] using the available translation and adaptation to Spanish [20]. This self-administered questionnaire consists of 2 parts: (1) for state anxiety (20 items) and (2) for trait anxiety (20 items), showing the Cronbach alpha coefficient range (.89-.95) for state anxiety and (.82-.91) for trait anxiety.
- Stress: Perceived stress was determined using a Visual Analogical Scale (VAS), where 0 is no stress and 10 is the greatest imaginable stress, similar to the analogical scales used by Girzadas et al [21] and Lima et al [22]. Indeed, the VAS has shown adequate construct validity in clinical environments in order to highlight differences in stress levels between two groups, showing acceptable correlations between the VAS and the anxiety domain (r=.66), depression domain (r=.45) and total score (r=.65) of the Hospital Anxiety and Depression Scale (HADS), and similar correlations with other tools applied to evaluate aspects of psychological distress, such as the Perceived Stress Scale (r=.64-.71) [23,24]. In addition, a test-retest reliability coefficient of .83 was reported for the perceived stress VAS [25].
- Age and gender: This quantitative variable was modified to a qualitative variable by grouping data into two intervals—younger than 25 years and older than 25 years.

Phase I involved standardized theoretical-practical training. Participants received a 20-hour theoretical and practical training course in Vital CPR, following the recommendations of the ERC 2010. The course took place in a simulation environment of medium fidelity for the acquisition of competencies in teamwork, coordination, airway management, cardiac massage, electrical therapies, and the administration of medication. Phase II called for the familiarization, preparation of the simulation case scenario, and the measurement of basal variables. At the onset of the standard training period, the students received a workshop for familiarization in high-fidelity simulation environments where they were able to practice on a high-fidelity simulation mannequin with physiological responses (Meti-man, CAE-LINK, Sarasota, FL, USA). One week before, the case was sent to students together with the patient history, the teaching objectives of the simulation and the supporting documentation based at best evidence practice, following the recommendations by Wilford and Doyle [16] and Lioce et al [17] for the design of simulation experiences. All students were presented with the case of a patient aged 67 years, with heart failure. The patient was in a stable condition and had been admitted to a hospitalization unit. The patient could evolve to 3 stages according to the actions and care of the team: improvement, worsening, and cardiorespiratory arrest. All participants were divided into 2 work groups based on their experience in health environments. In Group 1 the students had no experience in real health care environments. In Group 2 the students had experience in real health care environments (eg, emergency technicians, lifeguards, nursing assistants, and radio-diagnosis technicians). Once the groups were formed, they were distributed into smaller groups of 4 students, and the roles were explained (the students were not notified of their specific role until the moment they entered the simulation). One month earlier, the measurement of the following physiological, as well as psychological resting values, took place (study baseline measurements) as described in Textbox 1.

Phase III involved the development of clinical simulation scenarios (Cases 1-2) and measurement of variables. Roles were randomly assigned in a controlled environment, preserving both the intimacy as well as the confidentiality of the data, and all variables were measured immediately before (pre) and after (post) Cases 1 and 2. The students subsequently underwent the corresponding defusing and debriefing sessions. One week later, the students participated in a second case (Case 2) involving the same patient, undergoing the same measurements. Subsequently, a further debriefing session took place.

### Data Analysis

Data analysis was performed using the SPSS v.20.0 statistical software. Within the descriptive analysis of data, the categorical variables are expressed as the percentage, whereas the continuous variables are displayed as the mean and standard deviation. The Kolmogorov-Smirnov test was performed to evaluate the normality in the distribution of variables. When normality was verified, the Student’s t-test for related samples was applied to both groups. Statistical significance was set at *P*<.05. Reliability and validity were ensured by using established tools and approved equipment for testing purposes, as reported above.

## Results

### Descriptive Data

The final sample comprised of 107 students included 95 (88.8%) women and 12 (11.2%) men. Regarding the age of the participants, 97 were below 25 years of age (90.7%) whereas 10 (9.3%) were older than 25. Regarding the students’ professional experience in health care environments, 44 (41.2%) participants were experienced, whereas 63 (58.8%) had no experience. To facilitate the interpretation of the remaining results, these are presented below, for each of the comparisons performed, according to participants’ experience.

### Physiological and Psychological Variations Compared to Baseline Data

The normality of all variables was confirmed using the Kolmogorov-Smirnov test. Therefore, the Student’s t-test for related samples was applied to each group between the baseline moment and before Case 1 (pre-Case1). In Group 1 (without experience) a significant increase was observed in subjects before Case 1 compared to their baseline parameters of systolic blood pressure (SBP) with *P*<.001, diastolic blood pressure (DBP) with *P*=.001, HR (*P*<.001) and stress (*P*=.004), as shown in Table 1.

In Group 2 (with experience) a significant increase was observed in subjects prior to Case 1 compared to their baseline parameters for SBP (*P*<.001), DBP (*P*<.001), HR (*P*<.001), and without this change being observed in the stress levels (*P*=.10), as shown in Table 1.

### Physiological and Psychological Variations Between Pre- and Postparticipation in Simulation Scenarios

After confirming the normality of all the variables via the Kolmogorov-Smirnov test, the Student’s t-test for related samples was applied to each group to determine whether significant differences existed between different moments in time: (1) before Case 1 and after Case 1 (pre-Case 1/post-Case 1), (2) before Case 2 and after Case 2 (pre-Case 2/post-Case 2), and (3) before Case 1 and before Case 2 (pre-Case 1/pre-Case 2). Regarding Group 1 (without experience) the physiological parameters (Table 2), the SBP did not display significant changes during Case 1. However, a significant decrease occurred between the SBP before Case 1 and 2 (*P*=.01), as well as a significant increase during Case 2 (*P*=.001).

Regarding DBP, a significant decrease was observed (*P*=.02) between Cases 1 and 2, whereas within each of these cases there was no significant result (Table 2). In the case of HR, as with the SBP, a significant increase occurred during Case 2 (*P*<.001) as well as a significant decrease between Cases 1 and 2 (*P*=.01). Regarding Case 1, significant results did not occur between the moments in time (before and after), as shown in Table 2. In the case of the psychological parameters, a significant decrease in stress occurred before and after Case 1 (*P*=.003), as well as before Case 2, compared to Case 1 (*P*=.001). Regarding Case 2, we did not find significant changes between the different moments in time (before and after), as shown in Table 3.

Concerning state anxiety (Table 3), the subjects presented a significant decrease in Case 1 (*P*=.006), and during Case 2 (*P*=.002). However, significant differences were not found between Cases 1 and 2.

In Group 2 (with experience), regarding physiological parameters (Table 2), the SBP did not present significant changes in any of the cases under study. In the case of DBP, a significant decrease was found (*P*=.03) in Case 1 and between Cases 1 and 2 (*P*=.001). Regarding Case 2, significant results were not found between the different moments in time (before and after), as displayed in Table 2. Regarding HR values, subjects presented a significant increase of the same in Case 2. However, a significant decrease was not found between Cases 1 and 2 (*P*=.002). In Case 1, significant results did not occur when comparing the before and after values, as observed in Table 2. Concerning psychological parameters**,** only in Case 1 there was a significant decrease in stress (*P*=.002). There was no other significant effect in relation to this variable (Table 3). In the case of state anxiety (Table 3), participants presented a significant decrease in the same during Case 1 (*P*<.001). However, this was not significant in the remaining comparisons performed for this variable.

**Table 1:** The results of physiological resting variables and psychological variables measured at baseline and pre-Case 1, in both groups.

Variables		Baseline, mean (SD)	Pre-Case 1, mean (SD)	*P* value^a^
**Group 1 (without experience)**				
	SBP^b^ (mm Hg)	112.1 (7.8)	120.9 (12.0)	<.001
	DBP^c^ (mm Hg)	66.2 (6.2)	70.0 (9.0)	.001
	HR^d^ (bpm)	72.7 (11.7)	83.4 (19.4)	<.001
	Stress	5.4 (2.6)	6.2 (2.0)	.004
**Group 2 (with experience)**				
	SBP (mm Hg)	113.9 (9.00)	121.9 (10.2)	<.001
	DBP (mm Hg)	68.6 (6.0)	75.4 (8.0)	<.001
	HR (bpm)	77.0 (13.6)	86.1 (13.9)	<.001
	Stress	4.7 (2.8)	5.4 (2.4)	.10

^a^Statistical significance was set at *P*<.05.

^b^SBP: systolic blood pressure.

^c^DBP: diastolic blood pressure.

^d^HR: heart rate.

**Table 2:** Results of the physiological variables measured for both groups during the different study moments.

Variable			Mean (SD)	*P* value^a^
**Group 1 (without experience)**				
	**Systolic blood pressure (mm Hg)**			
		Pre-Case 1	120.9 (12.0)	N/A^b^
		Post-Case 1	120.5 (13.4)	N/A
		Variation during Case 1	–0.3 (12.1)	.83
		Pre-Case 2	117.7 (10.7)	N/A
		Post-Case 2	121.8 (11.2)	N/A
		Variation during Case 2	4.1 (9.5)	.001
		Pre-Case 1	120.9 (12.0)	N/A
		Pre-Case 2	117.7 (10.7)	N/A
		Variation between both cases	–3.2 (9.7)	.01
	**Diastolic blood pressure (mm Hg)**			
		Pre-Case 1	70.0 (9.0)	N/A
		Post-Case 1	70.3 (10.0)	N/A
		Variation during Case 1	0.4 (7.1)	.70
		Pre-Case 2	67.9 (9.2)	N/A
		Post-Case 2	68.7 (7.1)	N/A
		Variation during Case 2	0.8 (6.5)	.32
		Pre-Case 1	70.0 (9.0)	N/A
		Pre-Case 2	67.9 (9.1)	N/A
		Variation between both cases	–2.1 (7.1)	.02
	**Heart rate (bpm)**			
		Pre-Case 1	83.4 (19.4)	N/A
		Post-Case 1	84.8 (17.5)	N/A
		Variation during Case 1	1.3 (11.6)	.38
		Pre-Case 2	77.9 (12.7)	N/A
		Post-Case 2	84.8 (14.0)	N/A
		Variation during Case 2	6.9 (9.2)	<.001
		Pre-Case 1	83.4 (19.4)	N/A
		Pre-Case 2	77.9 (12.7)	N/A
**Group 2 (with experience)**				
	**Systolic blood pressure (mm Hg)**			
		Pre-Case 1	121.9 (10.2)	N/A
		Post-Case 1	122.0 (15.0)	N/A
		Variation during Case 1	0.1 (12.3)	.97
		Pre-Case 2	119.7 (12.7)	N/A
		Post-Case 2	121.0 (12.3)	N/A
		Variation during Case 2	1.3 (11.5)	.47
		Pre-Case 1	121.9 (10.2)	N/A
		Pre-Case 2	119.7 (12.7)	N/A
		Variation between both cases	–2.2 (9.7)	.15
	**Diastolic blood pressure (mm Hg)**			
		Pre-Case 1	75.4 (8.0)	N/A
		Post-Case 1	73.1 (9.8)	N/A
		Variation during Case 1	–2.3 (6.8)	.03
		Pre-Case 2	71.7 (8.8)	N/A
		Post-Case 2	72.3 (7.8)	N/A
		Variation during Case 2	0.7 (7.5)	.57
		Pre-Case 1	75.4 (8.0)	N/A
		Pre-Case 2	71.7 (8.8)	N/A
		Variation between both cases	–3.8 (6.7)	.001
	**Heart rate (bpm)**			
		Pre-Case 1	86.1 (13.9)	N/A
		Post-Case 1	84.9 (14.4)	N/A
		Variation during Case 1	–1.2 (7.5)	.29
		Pre-Case 2	80.3 (15.2)	N/A
		Post-Case 2	84.5 (16.3)	N/A
		Variation during Case 2	4.2 (8.3)	.002
		Pre-Case 1	86.1 (13.9)	N/A
		Pre-Case 2	80.3 (15.2)	N/A

^a^Statistical significance was set at *P*<.05.

^b^N/A: not applicable.

**Table 3:** Stress levels, measured using the visual analogue scale and values of state anxiety measured using the State-Trait Anxiety Inventory (STAI)-E scale for both groups, during the different study moments.

Variable			Mean (SD)	*P* value^a^
**Group 1 (without experience)**				
	**Stress**			
		Pre-Case 1	6.2 (2.0)	N/A^b^
		Post-Case 1	5.1 (2.7)	N/A
		Variation during Case 1	–1.1 (2.8)	.003
		Pre-Case 2	5.6 (2.3)	N/A
		Post-Case 2	5.1 (2.6)	N/A
		Variation during Case 2	–0.5 (2.7)	.17
		Pre-Case 1	6.2 (2.0)	N/A
		Pre-Case 2	5.6 (2.3)	N/A
		Variation between both cases	–0.7 (2.0)	.01
	**State Anxiety**			
		Pre-Case 1	25.4 (10.0)	N/A
		Post-Case 1	20.9 (11.7)	N/A
		Variation during Case 1	–3.9 (11.8)	.006
		Pre-Case 2	23.5 (11.6)	N/A
		Post-Case 2	18.6 (11.7)	N/A
		Variation during Case 2	–4.4 (11.0)	.002
		Pre-Case 1	25.4 (10.0)	N/A
		Pre-Case 2	23.5 (11.6)	N/A
		Variation between both cases	–1.9 (10.0)	.26
**Group 2 (with experience)**				
	**Stress**			
		Pre-Case 1	5.4 (2.4)	N/A
		Post-Case 1	4.4 (2.5)	N/A
		Variation during Case 1	–1.0 (2.0)	.002
		Pre-Case 2	4.9 (2.6)	N/A
		Post-Case 2	4.5 (2.7)	N/A
		Variation during Case 2	–0.4 (1.7)	.18
		Pre-Case 1	5.4 (2.4)	N/A
		Pre-Case 2	4.9 (2.6)	N/A
		Variation between both cases	0.5 (2.0)	.09
	**State Anxiety**			
		Pre-Case 1	23.7 (9.1)	N/A
		Post-Case 1	17.6 (10.2)	N/A
		Variation during Case 1	–6.1 (9.4)	<.001
		Pre-Case 2	20.8 (11.2)	N/A
		Post-Case 2	19.4 (12.6)	N/A
		Variation during Case 2	–1.4 (8.0)	.28
		Pre-Case 1	23.7 (9.1)	N/A
		Pre-Case 2	20.8 (11.2)	N/A
		Variation between both cases	–2.9 (8.7)	.10

^a^Statistical significance was set at *P*<.05.

^b^N/A: not applicable.

## Discussion

### Principal Findings

Different studies performed with both animals and humans have demonstrated the influence of hormones from the adrenal cortex upon the levels of stress, anxiety and physiological changes, and their relationship with learning. This demonstrates the influence of these hormones on memory and the individual perception of stress, together with its variations, before and after, any stressful situations [26-28]. On the other hand, as previously commented, high-fidelity simulation has a very high approximation accuracy to reality, by introducing the nursing student to realistic situations and contexts, similar to those that occur while providing care and which generate a high level of responses, both physiological as well as psychological and pedagogical. The results of our research indicate that the students who participated in the clinical simulation scenario began with values of BP, HR [29], general academic self-efficacy [30] and trait anxiety [20] which were within normal expected parameters in these age ranges.

### Physiological Values

In students without experience**,** a statistically significant increase of SBP, DBP, and HR was observed immediately before students participated in the first case (Case 1) compared with their baseline levels. This coincides with the work by Muller et al [31]. This increase seems to be related to factors such as uncertainty, the development of the case, the type of role, the management of the material, and the anticipatory anxiety. Nonetheless, these values tend to return to baseline values after debriefing and participation in a new clinical simulation scenario in line with research conducted to date by Dyer and Byrne [32] and Megel et al [33].

After participation in the second case (Case 2-students without experience), there was a significant decrease in all the physiological parameters measured before facing the second case, which is indicative of lesser stress and a greater level of adaptation, similar to the data collected by Bong et al [34]. This finding may be related to the perception of self-efficacy students have once they have been able to practice their skills during the first case and after a debriefing, during which they receive feedback on their skills within the simulation context. As noted by Bandura [35], the biological system is activated according to the belief that one has of his or her own ability to perform a task. Nonetheless, an increase in the vital signs was observed in students without experience, these changes were significant for the SBP and HR, coinciding with subjects with experience. This data, considering that it is the second experience, may demonstrate a more significant implication or physical effort in the case as these changes are not accompanied by an increase in the levels of stress (Table 3), as manifested by the students at the end of the exercise via an open survey designed for gathering students’ opinions regarding the simulation experience.

In the case of students with experience*,* the tendency of the vital signs is the same for students without experience. Initially, there is a significant increase in their baseline vital signs, which is probably related to the same causes mentioned in the group without experience. It is interesting to note that this group began with lower baseline levels, especially regarding stress and anxiety (Tables 1 and 3). Perhaps this variation is due to past professional experiences, which enable these students to view the context in a more familiar and less threatening way, thus providing them with a greater ability to adapt, as reflected in work by Karabacak et al [36]. This may be because they have acquired competencies due to vicarious learning, additional verbal information, and observation in their work context, as well as the perception of self-efficacy based on experience in a real health context.

When the students with experience participated in the second case (Case 2), an increase in HR was found (as occurred the group without experience). This was possibly related to the additional implication and effort and not due to an increase in stress, as previously noted. Furthermore, a decrease in all values was observed, which was statistically significant in the case of the DBP and the HR values, which also occurred in the group without experience, in accordance with the work by Dworkin et al [37], McKay et al [38] and DeMaria et al [39]. According to these studies, HR is a stable and reliable marker of the neuroendocrine response in situations of stress and is used with a greater frequency than the measurement of BP, as the latter is modified by many factors including instrumental complications for measurement of the same during a clinical simulation scenario.

Ultimately, an increase in the vital physiological signs was observed in both groups prior to participation in a first clinical simulation scenario, to later decrease, to a variable extent, according to experience and by serial participation in clinical simulation scenarios, which is in line with previous studies by Dyers and Byrne [32], Muller et al [31] and Bong et al [34].

### Psychological Values

In both groups and measurements, both pre-Case 1 compared to its baseline, and measurements taken post-Case 1, post-Case 2, and pre-Case 1- pre-Case 2, a decrease in the levels of stress and anxiety was observed, which is a finding that is also described by Henrich et al [40]. A more detailed analysis of our findings shows that among students without experience, the stress levels, before Case 1, were significantly higher than at baseline. Indeed, these were even higher than in students with experience. This is a finding that, as described by McGuire and Lorenz [41], may be related to the emotional state when facing an unknown situation with a certain level of uncertainty and insecurity. This is reflected in the activation of the sympathetic system which, in this case, seems to tend towards an adaptive-constructive process, as these stress levels decrease after the first case, this being a significant decrease. After participation in the second case, the level of stress decreased. However, this is not statistically significant, probably related to the fact that, in this case (Case 2), the students displayed lower levels of stress thanks to their participation in the first case and having debated the same.

Concerning state anxiety, a significant decrease was also found. We can postulate that the measurement of stress is related to a specific act (a real threat) and that, once this has ceased, the levels of stress decrease much faster. This is in contrast with state anxiety which depends on many personal factors, such as the effective situation at that time, the perception of a safe environment in contrast to the real context or findings of making a mistake while being observed as described by Khadivzadeh and Erfanian [42]. Similarly, the levels of stress and anxiety compared pre-Case 1 and pre-Case 2 decreased. This may suggest, as previously mentioned, a process of familiarization with the simulator and teamwork, in line with findings by Allan et al [43]. Among students with experience and at all assessments, a decrease in stress and anxiety was found which, in some cases, was statistically significant, as this group began with lower baseline values of stress and anxiety. Nonetheless, and despite the uncertainty, the feeling of being observed or the management of equipment, the fact these students had some prior related experience could favor their reduced anxiety in a simulation context compared to reality, which coincides with recent findings [13].

### Future Research Lines

From the teaching point of view, it would be interesting to devise proper practice guidelines to give students tools for being able to face clinical high-fidelity simulation experiences, in which a greater familiarization of students without experience is included, as well as ensuring students are gradually introduced to these methods. We believe it would be useful to perform studies on serial participation in clinical simulation scenarios to verify whether the levels of anxiety or stress show progression. Also, it would be useful to research whether markers of sympathetic activation, such as cortisol in saliva, alpha-amylase or perspiration can be determined to confirm the positive or negative influence of participation in clinical simulation scenarios.

### Limitations

It is essential to consider the sample size, although, to our knowledge, according to our literature search, this study has the highest number of subjects to date. On the other hand, we cannot affirm that the differences obtained between groups are only due to experience, as age may have a substantial influence upon vital signs, as well as aspects regarding the students’ personality, self-esteem, or fear of evaluation by peers. Therefore, a more significant sample is needed in future studies to perform a multivariate analysis. Also, the consecutive sampling method may be another limitation which should be considered in future studies.

### Conclusion

Participation in clinical simulation scenarios influences students both on a physiological and psychological level. In all students under study, vital signs increased before participation in a clinical simulation scenario, especially the heart rate. Furthermore, an increase in stress levels was observed, as well as in anxiety before their first simulation case. Both study groups (students with and without experience), demonstrated a decrease in the vital signs and levels of stress/anxiety, possibly related to the effects of participating in the case study scenarios as well as the debriefing sessions and subsequent simulation sessions, which suggests a positive adaptive process. Possibly, the incorporation of a simulation designed with different levels of complexity and realism, throughout the courses, helps to normalize vital signs and stress levels to figures close to normality before participation of students in these teaching methods.

## Abbreviations

BP: blood pressure
CPR: cardiopulmonary resuscitation.
DBP: diastolic blood pressure
ERC: European Resuscitation Council
HADS: Hospital Anxiety and Depression Scale
HFCS: high-fidelity clinical simulation
HR: heart rate
SBP: systolic blood pressure
